# *KIR*, *LILRB* and their Ligands’ Genes as Potential Biomarkers in Recurrent Implantation Failure

**DOI:** 10.1007/s00005-017-0474-6

**Published:** 2017-05-18

**Authors:** Izabela Nowak, Karolina Wilczyńska, Jacek R. Wilczyński, Andrzej Malinowski, Paweł Radwan, Michał Radwan, Piotr Kuśnierczyk

**Affiliations:** 1 Department of Clinical Immunology, Laboratory of Immunogenetics and Tissue Immunology, Ludwik Hirszfeld Institute of Immunology and Experimental Therapy, Polish Academy of Sciences, Rudolfa Weigla 12, 53-114 Wrocław, Poland; 2 Department of Gynecology and Gynecologic Oncology, Polish Mothers’ Memorial Hospital-Research Institute, Rzgowska 281/289, 93-338 Łódź, Poland; 3 Department of Surgical, Endoscopic and Oncologic Gynecology, Polish Mothers’ Memorial Hospital-Research Institute, Rzgowska 281/289, 93-338 Łódź, Poland; 4 Department of Reproductive Medicine, Gameta Hospital, Rudzka 34/36, 95-030 Rzgów, Poland; 5 Biogeno, Regional Science-Technology Centre, Podzamcze 45, 26-060 Chęciny Kielce, Poland

**Keywords:** Recurrent implantation failure, In vitro fertilization, Polymorphism, KIR, LILRB, HLA

## Abstract

Reproductive failure in humans is a very important social and economic problem, because nowadays women decide to conceive later in life and delay motherhood. Unfortunately, with increasing age they have less chance for natural fertilization and maintenance of pregnancy. Many of them need assisted reproductive technology. Approximately 10% of women after in vitro fertilization-embryo transfers experience recurrent implantation failure (RIF). Multiple factors may contribute to RIF, including oocyte and sperm quality, parental chromosomal anomalies, genetic or metabolic abnormalities of the embryo, poor uterine receptivity, immunological disturbances in the implantation site, and some gynecologic pathologies such as endometriosis, uterine fibroids, hydrosalpinx and endometrial polyps. Moreover, the procedure of in vitro fertilization itself could adversely influence the implantation. Nowadays, many studies are focused on the role of natural killer (NK) cells in normal and pathologic pregnancy because NK cells constitute the dominant cell population in the endometrium and they come in close contact with the allogeneic extravillous trophoblast cells in early pregnancy decidua. The majority of these cells are of CD56^bright^ phenotype. These cells can express killer immunoglobulin-like receptors (KIRs), which, upon recognition of HLA class I molecules (HLA-C and HLA-G) on trophoblasts, may either stimulate or inhibit NK cells to produce soluble factors, and display low cytotoxicity necessary for maintenance of the allogeneic embryo and fetus in the next steps of pregnancy. Moreover, some members of the leukocyte immunoglobulin-like receptor (LILR) family, also named ILT (immunoglobulin-like transcript), are present in the human placenta. LILRB1 (ILT2) was described mainly on stromal cells, while LILRB2 (ILT4), in addition to stromal cells, was also found around vessels in the smooth muscle layer. In this review we focus on the possible role of polymorphism of KIR, LILRB and their ligands (HLA-C, HLA-G) in susceptibility to recurrent implantation failure, which could serve as diagnostic biomarkers of this disease.

## Introduction

Reproductive failure in humans is a very important social and economic problem, because nowadays women decide to conceive later in life and delay motherhood. Unfortunately, with increasing age they have less chance for natural fertilization and maintenance of pregnancy. Many of them need assisted reproductive technology (ART). Regrettably, clinicians have observed increasing numbers of cases with recurrent implantation failure (RIF) after in vitro fertilization-embryo transfers (IVF-ETs). It is estimated that approximately 10% of women following IVF treatment will experience this particular problem (Koot et al. 2012).

Normal human reproduction is an inefficient process, because only about 20–25% of conceptive matings appear to result in a live birth (Clark 2003; Polanski et al. 2014; Sharkey and Macklon 2013). However, only 5–15% of failed pregnancies are clinically seen. This indicates that most pregnancy failures are preclinical. Most pregnancy wastage is caused by “abnormal” embryos, as we know that there are significant chromosome abnormalities in 70% of sporadic abortions. Moreover, epidemiological data indicate that an additional 27% of “normal” embryos are lost at or after the time of implantation, resulting in the situation where in healthy couples approximately half of all human embryo implantations (both abnormal and normal embryos) result in failed pregnancy (Clark 2003). Before the era of IVF the fact of early pregnancy loss was not commonly known. IVF added the ability to compartmentalize the treatment process so that it became possible to know when an embryo was transferred and if an implantation occurred. Hence, recurrent implantation failure became a possible clinically identifiable phenomenon. However, the definition of RIF still depends on the clinical approach to the problem (Koot et al. 2012; Polanski et al. 2014; Vlachadis et al. 2014; Simon and Laufer 2012). The first definition commonly used describes RIF as a lack of pregnancy after at least three embryo transfers with good quality of embryos (Coughlan et al. 2014; Koot et al. 2012; Toth et al. 2011). However, defining RIF as three unsuccessful IVF attempts actually defines RIF in terms of failed IVF cycles and does not address the issue of implantation rates. Therefore, a second method of defining RIF uses the number of embryos transferred without achieving a pregnancy. According to some opinions, RIF should be defined as having failed to achieve a viable pregnancy if more than 12 embryos have been transferred (Simon and Laufer 2012). However, most IVF centers now have better implantation rates, partly because of improved quality of culture media used for the IVF procedure. Such centers define RIF as a lack of clinical pregnancy after the transfer of four or more good-quality embryos in a minimum of three fresh or frozen cycles in a woman under the age of 40 years (Coughlan et al. 2014) or two consecutive cycles (Polanski et al. 2014).

Multiple factors may contribute to RIF, including the woman’s age, oocyte and sperm quality, parental chromosomal anomalies, genetic or metabolic abnormalities of the embryo, poor uterine receptivity, and immunological disturbances in the implantation site. Some gynecologic pathologies, such as endometriosis, uterine fibroids, hydrosalpinx and endometrial polyps, could negatively affect the implantation rate. Moreover, the process of preparation for ART itself could adversely influence the implantation rate. Finally, other factors such as lifestyle, i.e., smoking, alcohol consumption, and obesity, could impair the likelihood of reproductive success (Cakmak and Taylor 2011; Coughlan et al. 2014; Das and Holzer 2012; Koot et al. 2012; Penzias 2012).

## KIRs and their Ligands

Nowadays, many studies are focused on the role of natural killer (NK) cells in normal and pathologic pregnancy, as well as the possible role in implantation failure after IVF (Miko et al. 2010; Quenby and Farquharson 2006). NK cells constitute the dominant cell population in the preimplantation endometrium (Tuckerman et al. 2010), and they come in close contact with the allogeneic extravillous trophoblast cells in early pregnancy decidua. The majority of these cells are of CD56^bright^ phenotype. These cells can express killer immunoglobulin-like receptors (in brief KIRs), which, upon recognition of HLA class I molecules (HLA-C and HLA-G) on trophoblasts, may either stimulate or inhibit NK cells to produce soluble factors, and display low cytotoxicity necessary for the maintenance of a semiallogeneic fetus (Augusto and Petzl-Erler 2015; Makrigiannakis et al. 2011; Moffett and Shreeve 2015; Zhang et al. 2016).

KIR receptors (killer cell immunoglobulin-like receptors) are members of the immunoglobulin superfamily. They are expressed on the surface of NK cells and some T lymphocytes. KIRs are characterized by two (KIR2D, D1-D2 or D0-D2) or three (KIR3D, D0-D1-D2) extracellular domains and the length of the cytoplasmic tail. Those with long cytoplasmic tails and possessing immunoreceptor tyrosine-based inhibitory motifs (ITIMs) are named KIR2DL/KIR3DL. Those with short cytoplasmic tails containing a positively charged amino acid residue in the transmembrane region are named KIR2DS/KIR3DS. KIRs with short cytoplasmic tails due to the complex with DAP12 (an adaptor signaling molecule with immunoreceptor tyrosine-based activating motif—ITAM) cause activation of NK cells upon interaction with their ligands, in contrast to KIR2DL and KIR3DL, which cause inhibition (Augusto and Petzl-Erler 2015; Carillo-Bustamante et al. 2016; Moffett and Colucci 2015).

**Fig. 1:**
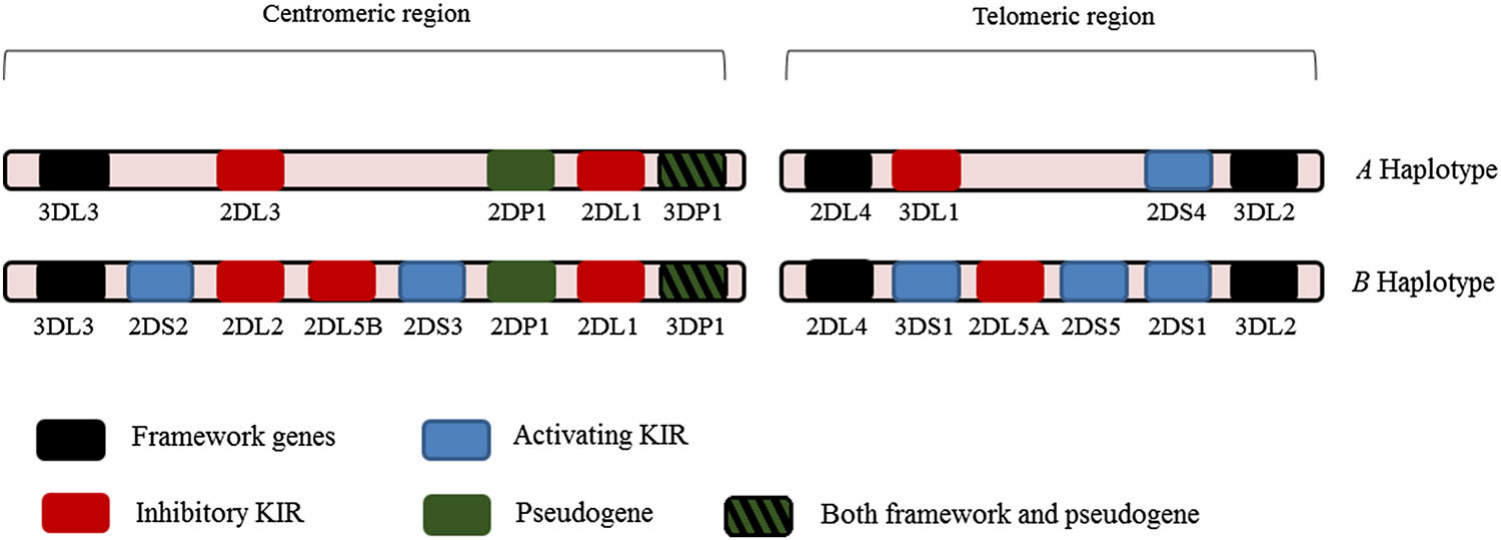
Schematic diagram of centromere and telomere KIR haplotypes

*KIR* genes exhibit extensive haplotypic polymorphism. Individuals differ in both the number and kind (activating vs. inhibitory) of *KIR* genes. *KIR* genes can be divided into two major haplotypes—A and B. Both haplotypes have four common conserved framework genes—*KIR3DL2*, *KIR3DL3*, *KIR3DP1* (P refers to pseudogene), and *KIR2DL4*. The simpler group A haplotype is generally non-variable and comprises a fixed gene content of inhibitory genes—*KIR2DL1*, *2DL3* and *3DL1* with *2DS4* the single activating gene. Group B haplotypes contain a variable gene combination, but tend to encode more activating *KIR*s (Hammond et al. 2016; Middleton and Gonzelez 2010; Parham et al. 2012). Figure 1 shows organization of *KIR* haplotypes.

Each KIR has a subgroup of HLA class I allotypes as its ligand. KIR2DL1 and KIR2DL2/3 recognize distinct HLA-C allotypes, based on polymorphisms at positions 77 and 80 in the α1-domain of the HLA heavy chain. For example, KIR2DL1 binds HLA-Cw2, HLA-Cw4, HLA-Cw5, and HLA-Cw6 (called C2), whereas KIR2DL2 and KIR2DL3 bind to HLA-Cw1, HLA-Cw3, HLA-Cw7, and HLA-Cw8 (called C1). KIR3DL1 ligands are HLA molecules sharing the Bw4 epitope representing around 50% of human HLA-B alleles, and KIR3DL2 binds HLA-A3 and HLA-A11. KIR2DL1 has a high affinity for C2 allotypes, whereas KIR2DL2/3 has a high affinity for C1 (albeit lower than that of KIR2DL1 for C2), but also for a few members of HLA-B alleles, and a low affinity for C2 allotypes. The interactions of activating KIR receptors with HLA ligand are not completely known. The study by Varbanova et al. (2016) showed that KIR3DS1 recognizes HLA-B*2705. Another study demonstrated that HLA-F is a ligand of KIR3DS1 (Burian et al. 2016). Moreover, some studies revealed that KIR2DS2 binds to HLA-A*11:01 (Liu et al. 2014). Table 1 summarizes all known *KIR* and *LILRB* genes and their ligands.

**Table 1:** *KIR* genes and *HLA* ligands

KIR	Ligand	References
2DL1	HLA-C C2: C*02, C*04, C*05, C*06	Varbanova et al. (2016)
2DL2	HLA-C C1: C*01, C*03, C*07, C*08 Some HLA-C C2: C*0501, C*0202, C*0401 Some HLA-B: B*4601, B*7301	Varbanova et al. (2016)
2DL3	HLA-C C1: C*01, C*03, C*07, C*08 Some HLA-C C2: C*0501, C*0202 Some HLA-B: B*4601, B*7301	Varbanova et al. (2016)
2DL4	HLA-G	Saunders et al. (2015)
2DL5A and 2DL5B	Unknown	Ivarsson et al. (2014)
2DS1	HLA-C C2: C*02, C*04, C*05, C*06	Kennedy et al. (2016)
2DS2	HLA-A*1101 HLA-C C1 (weak)	Liu et al. (2014), Ivarsson et al. (2014)
2DS3	Unknown	Varbanova et al. (2016)
2DS4	Some HLA-A: A*1102 Some HLA-C: C*0501, C*1601, C*0202	Varbanova et al. (2016), Ivarsson et al. (2014)
2DS5	Unknown	Varbanova et al. (2016)
3DL1	Some HLA-A and B expressing Bw4 epitope HLA-B: B*08, B*27, B*57, B*58 HLA-A: A*24, A*23, A*32	Augusto and Petzl-Erler (2015), Saunders et al. (2015)
3DS1	HLA-Bw4 epitope: HLA-B*5701 HLA-F	Kennedy et al. (2016), Saunders et al. (2015), Varbanova et al. (2016), Burian et al. (2016)
3DL2	Some HLA-A: A*03, A*11 HLA-B27 HLA-F	Augusto and Petzl-Erler (2015) Shaw et al. (2014) Goodridge et al. (2013)
3DL3	Unknown	Ivarsson et al. (2014)
LILRB1 (ILT2)	All HLA class I	Kang et al. (2016)
LILRB2 (ILT4)	All HLA class I	Kang et al. (2016)

Polymorphism of *KIR* and *HLA* affects NK cell reactivity and susceptibility to various diseases, including gynecological disorders such as recurrent miscarriage, preeclampsia (Hiby et al. 2004, 2008, 2010), and perhaps recurrent implantation failure (Falco et al. 2013; Parham et al. 2012). In 78% of patients with more than five unsuccessful IVF treatments or embryo transfers, killer-cell immunoglobulin-like receptor typing revealed the lack of three activating receptors (2DS1, 2DS3 and 3DS5). In this group of patients, where *KIR* testing indicates the presence of defects in maternal-embryonic implantation communication, the use of granulocyte colony-stimulating factor is an extremely promising additional method of treatment (Würfel et al. 2010). In addition, Alecsandru et al. (2014) observed significantly higher rates of early miscarriage per cycle after double embryo transfer (DET) with the patient’s own oocytes in mothers with the KIR AA haplotype (22.8%) compared with KIR BB haplotype mothers (11.1%). Moreover, decreased live birth rates per cycle were found after DET of donated oocytes in mothers with the KIR AA haplotype (7.5%) compared with those with the KIR AB (26.4%) and KIR BB (21.5%) haplotypes (*P* = 0.006). However, there were no significant differences for pregnancy, miscarriage and live birth rates per cycle among those with maternal KIR AA, AB and BB haplotypes after single embryo transfer with the patient’s own or donated oocytes. In turn, Varla-Leftherioti et al. (2007, 2010), in relatively small numbers of RIF couples (40 and 61, respectively), observed a lower percentage of patients with the most inhibiting combination of genes (KIR2DL1-HLA-C2) in comparison to controls. Moreover, they did not detect this combination at all in women with six implantation failures after IVF.

## KIR2DL4 and LILRB as Competitors to HLA-G Binding

It should be highlighted that KIR2DL4 is the most unusual among KIR receptors because of its structure, cellular localization, expression, signaling, and ligand specificity. It does not contain a D1 domain, but it has D0–D2 structure. This receptor possesses a single ITIM in its cytoplasmic tail and a positively charged arginine in the transmembrane region, suggesting capacity for both activation and inhibition (Moradi et al. 2015). To transduce stimulatory signals KIR2DL4 recruits the FcεR-γ chain, instead of DAP12 as in other activating KIRs. KIR2DL4, constitutively expressed by all NK cells and some T cells at the transcriptional level, is variable in its surface expression. KIR2DL4 is expressed at the surface of NK CD56^bright^, and it occurs on the majority of decidua-placental NK cells, but not peripheral NK CD56^dim^. KIR2DL4 also localizes in early endosomes and binds to the non-classical class I protein HLA-G (Rajagopalan et al. 2001; Rajagopalan and Long 2012). In its gene structure we can distinguish alleles with either nine or ten consecutive adenines in exon 7 (rs11410751), which encodes the transmembrane domain. The deletion of one adenine in the 9A allele results in a frame-shift and the creation of either a protein with a truncated cytoplasmic tail or one lacking the transmembrane region; thereby it causes a lack of KIR2DL4 expression at the cell surface. In turn, the 10A alleles encode receptors which can be expressed at the cell surface (Goodridge et al. 2009; Nowak et al. 2015). In view of the *KIR2DL4* polymorphism we may speculate that it may have an influence on the interaction of decidual NK cells with HLA-G expressed on the trophoblastic cells. It is worth underlining that the *KIR2DL4* gene is present in almost all people. It has been reported that a few women worldwide who gave birth to healthy children did not possess *KIR2DL4* (Gómez-Lozano et al. 2003; Nowak et al. 2011). This phenomenon indicates that many mechanisms and many cell types take part in the maintenance of immunological tolerance of the mother to the fetus; i.e., lack of KIR2DL4 may be compensated by the presence of respective LILRB1, which belongs to the LILR (leukocyte immunoglobulin-like receptor), also named as ILT (immunoglobulin-like transcript), family. These receptors, similar to KIRs, are encoded on chromosome 19 in the q13 region. To date, 13 receptors belonging to this family have been described. They are different in structure, expression, cellular appearance, and function. LILRA (activating) and LILRB (inhibitory) receptors have wider distribution than KIR, as they are expressed on myelomonocytic cells, lymphocytes, NK cells, dendritic cells, macrophages, B cells, and placental stromal cells (Hudson and Allen 2016; Kang et al. 2016). Their function is also dependent on ITIM (for LILRB) and ITAM (for LILRA) sequences (Anderson and Allen 2009; Hudson and Allen 2016). Among LILRBs we can distinguish LILRB1 (ILT2) and LILRB2 (ILT4), which are both present in the human placenta. LILRB1 was observed mainly on stromal cells, while LILRB2, in addition to stromal cells, was also observed around vessels in the smooth muscle layer (McIntire et al. 2008). However, the same group in the next study did not confirm LILRB2 expression around vessels in the smooth muscle layer (Hunt and Clavellina 2010). Moreover, a higher expression level of ILT2 and ILT4 transcripts was observed in control deciduas than that in recurrent abortion deciduas. Inhibitory function of ILT2 and ILT4 may counter the activating signal of KIR2DL4 when binding to HLA-G localized on the trophoblast (Yan et al. 2007). In another study performed by Djurisic et al. (2015) KIR2DL4 and LILRB1 expression was upregulated on uterine NK cells. They also found a correlation between uterine soluble HLA-G (sHLA-G) and the fraction of KIR2DL4 positive uterine NK cells. The authors hypothesize that the phenotype of uterine NK cells may be influenced by HLA-G on trophoblast cells and by sHLA-G in the uterus.

Moreover, a study performed by our group on the *KIR*, *LILRB1* and *HLA*-*G* association with spontaneous miscarriage indicated that polymorphism of partners in *KIR2DL4* could be associated with susceptibility to miscarriage of their women (Nowak et al. 2016). Whether the scenario in which the paternal *KIR2DL4* allele inherited by the embryo could have an impact on NK cell responses and, therefore, could have an influence on the grade of implantation remains to be elucidated. It should be mentioned that the expression of KIR2DL4 and LILRB1 receptors in both primary trophoblasts (first trimester) and trophoblastic cell lines (JAr and JEG-3) has been described by Guo et al. (2013). Moreover, these receptors were functional, as trophoblast invasion was induced by binding sHLA-G to KIR2DL4 and LILRB1. However, we do not know when exactly the expression of KIR2DL4 and LILRB receptors appears for the first time in embryonic development.

In addition, KIR2DL4 possesses an alternative ligand, heparan sulfate/heparin glycosaminoglycans (GAGs) (Brusilovsky et al. 2013, 2014). Interactions of KIR2DL4 and GAG can affect receptor function. Therefore, we may hypothesize that trophoblast KIR2DL4 inherited from the father may interact with GAG-containing proteoglycans, and that this interaction may be affected by *KIR2DL4* polymorphism. Moreover, many patients with spontaneous miscarriage and an increased risk of thrombosis receive low molecular weight heparin to reduce blood clotting. A very interesting study would, therefore, be to analyze the impact of *KIR2DL4* genotype on the outcome of pregnancy in patients treated with various doses of heparin.

In PubMed we could not find studies on the role of LILRB polymorphism with susceptibility to RIF, although there is a rationale for this. LILRB1, which contains an immunoreceptor tyrosine-based switch motif in its cytoplasmic region, may act as an activating receptor, but it may also exert inhibitory functions. The activating role of LILRB1 has also been suggested by Li et al. (2009). They cultured cells of homodimer-HLA-G transfectants 721.221 with human decidual CD14^+^ macrophages or NK cells isolated from terminated first trimester pregnancies and cross-linked them with anti-LILRB1 and anti-KIR2DL4 antibodies. This resulted in upregulation of interleukin (IL)-6, IL-8 and tumor necrosis factor-α transcripts. It should be noted that LILRB1 binds more strongly to HLA-G than to classical HLA class I molecules and HLA-G dimer induces more efficient LILRB1 signaling than the monomeric form (Shiroishi et al. 2003, 2006a, b).

HLA-G belongs to the non-classical class I human leukocyte antigens, and it is characterized by limited tissue distribution and lower polymorphism. Only 53 alleles and 18 proteins of this molecule have been identified (IPD—IMGT/HLA database; accessed January 2017). The most polymorphic sites of *HLA*-*G* were found in the promoter and 3-untranslated (3′UTR) gene, resulting in diversification of HLA-G expression. The *HLA*-*G* gene, due to alternative splicing of its transcript, encodes seven proteins: four are membrane-bound (HLA-G1 to HLA-G4), while three (HLA-G5 to HLA-G7) are soluble proteins (Menier et al. 2010). In pregnancy, expression of HLA-G is determined by the kind of trophoblast and stage of pregnancy progression. HLA-G membrane-bound molecules are presented by all subpopulations of extravillous trophoblasts. Soluble isoforms (HLA-G5-7) were detected in maternal-fetal circulation, amniotic fluid, and in all trophoblasts (McIntire et al. 2008). Soluble HLA-G can be detected in plasma or serum not only from pregnant women, but also from non-pregnant ones and from men, but the concentration of sHLA-G is 2–4 times higher in blood from pregnant than non-pregnant women. Moreover, sHLA-G levels are detected to be higher in the first trimester of pregnancy compared to the second and third trimesters (Rizzo et al. 2009). Finally, HLA-G expression was shown in human embryonic stem cells, human oocytes and preimplantation embryos. Notably, HLA-G expression differed during development of the blastocyst, as was shown in confocal microscopy (Verloes et al. 2011). Soluble HLA-G was also found in day-2 embryos after intracytoplasmic sperm injection. Data from this multicenter study show that sHLA-G may be a marker in improving pregnancy outcome with the ability to reduce multiple pregnancies (Kotze et al. 2013). The study published by Dahl et al. (2014) showed a significantly higher concentration of sHLA-G in seminal plasma samples associated with homozygosity of a 14 base pair (bp) insertion/deletion (ins/del) polymorphism in the 3′UTR region of the *HLA*-*G* gene, where the assisted reproduction treatment was successful compared with men, where the ART did not result in pregnancy. If the placenta contains proteins, perhaps with immunoregulatory function, that were already present in the semen, then it might be possible that sexual exposure could be one of the mechanisms to acquire immunological tolerance of the mother to the embryo/fetus. Thus, studies should include both partners in a study on the role of depicted genes and sHLA-G in blood plasma. Also, the study of the 14 bp ins/del 3′UTR polymorphism is advisable because of the correlation of ins/ins genotype with lower mRNA production of HLA-G as opposed to del/del genotype, which is associated with high expression of HLA-G mRNA. This results in production of sHLA-G, namely individuals exhibiting the 14 bp del/del and 14 bp ins/del genotypes show higher levels of sHLA-G compared to the 14 bp ins/ins genotype (Martelli-Palomino et al. 2013).

To the best of our knowledge, polymorphism of *HLA*-*G* and its role in RIF have been investigated in only a few studies that obtained contradictory results. In the Danish population ins/ins 14 bp genotype in 3′UTR was associated with unsuccessful ART treatment (Hviid et al. 2004a, b). According to the report of Costa et al. (2012) on 25 Brazilian couples there were no differences in the frequency of ins/del 14 bp polymorphism between patients who underwent IVF and the control (fertile) group. However, researchers found an association of the haplotype consisting of the HLA-G*01:01:02a allele and T in the −1140 gene position and a 14 bp insertion with RIF. The same group of researchers in 2016 detected the haplotype HLA-G*01:01:01b/HLA-G*01:01:01 with significantly higher frequency in control groups (Costa et al. 2016). The study conducted in a Polish population by Sipak-Szmigiel et al. (2009) concerned the polymorphism of −725C > G in the promoter region and 14 bp ins/del polymorphism in 3′UTR of *HLA*-*G.* Researchers found increased frequency of ins/ins or ins/del genotype in 3′UTR of patients after IVF in comparison to fertile women. The frequency of the ins/ins or ins/del genotype was even increased to 90% in women who experienced five or more IVFs. However, after correction for multiple comparisons this result was not statistically significant, possibly because of the small size of the investigated group (50 couples after IVF-ET, and 71 control couples). The potential role of HLA-G in the success of IVF-ET has also been indicated by Lashley et al. (2014), who performed genotyping of women with RIF and their partners for HLA class I, HLA class II, HLA-G, and KIR alleles. Results were compared with those obtained from couples with successful embryo implantation after their first IVF procedure and normal fertile couples. A higher frequency of HLA-C2 and the 14 bp insertion in HLA-G was found in women with RIF in comparison to controls. The researchers concluded that these two genetic loci represent a risk factor which may affect the success of IVF.

## Concluding Remarks

Our hypothesis assumes that reproductive success depends on the immunological tolerance of the mother to the fetus. The balance of all activating and inhibiting signals between NK cells in the decidua and trophoblast is the most important factor and may have an influence on embryo implantation. We believe that investigation of the genetic background of receptors involved in this process—KIR, LILRB, HLA-C and HLA-G—may help in diagnostics of RIF and forecasting results of therapy, and may help in clarification of disease pathogenesis. However, studies should be performed on larger groups of patients and controls in homogenic populations. These studies should also contain two appropriate control groups: first, fertile couples who spontaneously conceive with no earlier spontaneous abortion or any other immunological and gynecological diseases and second, couples who underwent IVF, became pregnant and gave birth to a healthy child. As the ethnicity is important in the distribution of KIR, LILRB and HLA alleles in different populations, we suggest that more IVF centers from all over the world should be engaged in research on the recurrent implantation failure.

## Abbreviations

14 bp ins/del 3′UTR: 14 base pair insertion/deletion polymorphism in the 3′-untranslated
ART: Assisted reproductive technology
DAP12: An adaptor signaling molecule with immunoreceptor tyrosine-based activating motif
DET: Double embryo transfer
FcεR-γ: Gamma chain of receptor for the Fc region of immunoglobulin E
GAG: Glycosaminoglycan
HLA: Human leukocyte antigen
ILT: Immunoglobulin-like transcript
ITAM: Immunoreceptor tyrosine-based activating motif
ITIM: Immunoreceptor tyrosine-based inhibitory motif
IVF: In vitro fertilization
IVF-ET: In vitro fertilization-embryo transfer
KIR: Killer immunoglobulin-like receptor
LILR: Leukocyte immunoglobulin-like receptor
LILRA: Activating leukocyte immunoglobulin-like receptor
LILRB: Inhibitory leukocyte immunoglobulin-like receptor
NK cell: Natural killer cell
RIF: Recurrent implantation failure
sHLA-G: Soluble HLA-G
